# The Sources, Synthesis and Biological Actions of Omega-3 and Omega-6 Fatty Acids in Red Meat: An Overview

**DOI:** 10.3390/foods10061358

**Published:** 2021-06-11

**Authors:** Eric N. Ponnampalam, Andrew J. Sinclair, Benjamin W. B. Holman

**Affiliations:** 1 Animal Production Sciences, Agriculture Victoria Research, Department of Jobs, Precincts and Regions, Bundoora, VIC 3083, Australia; 2 Department of Nutrition, Dietetics and Food, Monash University, Clayton, VIC 3800, Australia; andrew.sinclair@monash.edu; 3 Centre for Red Meat and Sheep Development, NSW Department of Primary Industries, Cowra, NSW 2794, Australia; benjamin.holman@dpi.nsw.gov.au

**Keywords:** agricultural practices, animal production, nutrition, human health, fatty acid profile, fat digestion and absorption, consumer guidelines, preservation

## Abstract

The maximisation of available resources for animal production, food security and maintenance of human–animal wellbeing is important for an economically viable, resilient and sustainable future. Pasture and forage diets are common sources of short chain omega-3 (n-3) polyunsaturated fatty acids (PUFA), while grain-based and feedlot diets are common sources of short chain omega-6 (n-6) PUFA. Animals deposit n-3 and n-6 PUFA as a result of their direct consumption, as feeds or by synthesis of longer chain PUFA from short chain FA precursors in the body via desaturation and elongation processes. Research conducted over the last three decades has determined that the consumption of n-3 PUFA can improve the health and wellbeing of humans through its biological, biochemical, pathological and pharmacological effects. n-6 PUFA also play an important role in human health, but when consumed at high levels, are potentially harmful. Research shows that current consumption of n-6 PUFA by the human population is high due to their meal choices and the supplied food types. If consumption of n-3 PUFA from land- and marine-based foods improves human health, it is likely that these same food types can improve the health and wellbeing of livestock (farm animals) by likewise enhancing the levels of the n-3 PUFA in their circulatory and tissue systems. Modern agricultural systems and advanced technologies have fostered large scale animal and crop production systems. These allow for the utilisation of plant concentrate-based diets to increase the rate of animal growth, often based on economics, and these diets are believed to contribute to unfavourable FA intakes. Knowledge of the risks associated with consuming foods that have greater concentration of n-6 PUFA may lead to health-conscious consumers avoiding or minimising their intake of animal- and plant-based foods. For this reason, there is scope to produce food from plant and animal origins that contain lesser amounts of n-6 PUFA and greater amounts of n-3 PUFA, the outcome of which could improve both animal and human health, wellbeing and resilience to disease.

## 1. Introduction

The utilisation of available resources for resilient animal production systems, food security and maintenance of the health and wellbeing of human–animal population are important for the sustainability of future agriculture and food production. The use of available feed resources from cultivated pasture and natural range lands in animal production systems may be beneficial for the wellbeing of livestock. The same is true for those humans who consume milk, meat and offal from those animals, especially when compared to products from livestock fed commercially formulated feeds or low-quality forages that are deficient in nutrients. In many livestock production systems, pasture and forage diets are common sources of omega-3 (n-3) polyunsaturated fatty acids (PUFA). Grain-based and feedlot diets are, instead, common sources of omega-6 (n-6) PUFA.

**Figure 1:**
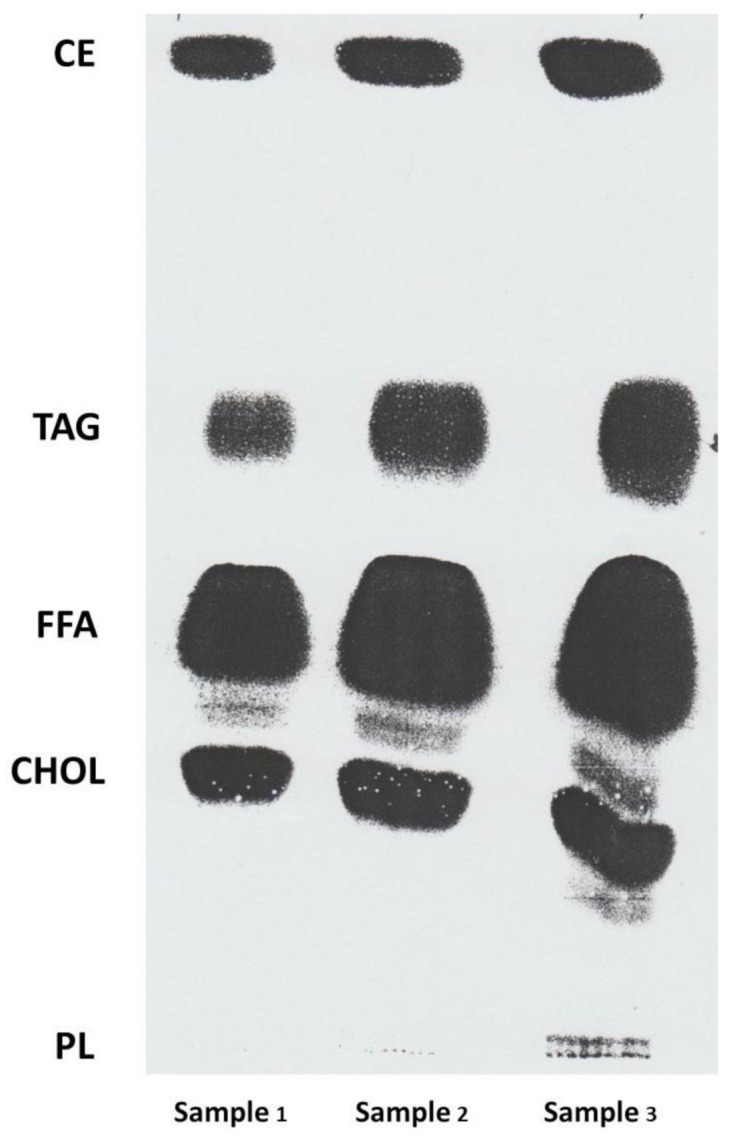
Lipid fractions of lyprinol (green-lipped mussel) separated by using a thin layer chromatography (TLC) technology. Lipid fractions were separated by thin layer chromatography (TLC) on silica gel plates (Silica gel 60H, Merck, Darmstadt, Germany). The solvent system for all TLC was petroleum spirit/diethyl ether/glacial acetic acid (85:15:2 by volume). Lyprinol (50 g) was made up in 1 mL of chloroform, and from this stock, 10 µL (sample 1), 20 µL (sample 2) and 30 µL (sample 3) were spotted as shown above. Lipid classes were visualised with fluorescein 5-isothiocyanate against TLC standard 18-5 (Nuchek Prep Inc, Elysian, MN). Lipid fractions identified from left top to bottom are cholesterol esters (CE), triacylglycerols (TAG), free fatty acids (FFA), cholesterols (CHOL) and phospholipids (PL).

It is well known that lipids (fats from animals and oils from plants) provide energy, nutrient mediation, signal transduction, disease prevention, insulation, cell membrane structure, and organ protection upon consumption. Lipids include triglyceride, phospholipid, cholesterol, cholesterol ester, free fatty acids (FFA), sphingomyelin subgroups and glycolipids. Not many animal or plant scientists understand the complexity of the lipid fraction and the differences between animal and plant tissues. For example, fat deposits in animal tissue are mainly triacylglycerols (TAG); muscle lipids contain TAG, cholesterol and phospholipids; plant leaf tissue lipids are mainly polar lipids (glycerophospholipids); oil seed lipids are mainly TAG. The different lipid fractions in plant and animal tissues can be separated and observed by using a thin layer chromatography (TLC) technology, an example is shown in Figure 1.

Previous research of both humans and animals, including livestock and companion animals, demonstrate that dietary background plays a major role on lipid metabolism, fatty acid (FA) synthesis and fat accretion in the body—more so than genetic or gender associated factors alone. The effects of dietary fat are contributed by both their energy concentration and the types of lipids present. Genetic effects in FA synthesis and accretion in farm animals has been shown to be associated with desaturase and elongation activity [1]. Genetics are also known to influence desaturase and elongase activity in humans [2]. There are many studies which refer to the influence of gender on PUFA synthesis in human (higher activity in females than males).

Fatty acids are classified as saturated or unsaturated. Unsaturated FA can be further delineated as monounsaturated (MUFA) and PUFA. Common dietary sources of PUFA include leafy vegetables, oilseed, nuts, meat, eggs and seafood. Characteristics of PUFA are their low melting point and liquid state when held at room temperature. Hence, these are often referred to as oils. It is of interest, therefore, that fat melting point has been applied to estimate the unsaturated FA content of non-liquefied fat deposits from, for example, beef and sheep meat [3,4]. There is substantive evidence to support the regular consumption of n-3 PUFA, as these are beneficial for growth, development, health and the welfare of humans and animals [5,6,7,8,9]. The n-3 PUFA alpha-linolenic acid (ALA, C18:3n-3) and the long chain derivatives eicosapentaenoic acid (EPA, C20:5n-3), docosapentaenoic acid (DPA, C22:5n-3) and docosahexaenoic acid (DHA, C22:6n-3) have each been reported to play a role in the prevention of cardiovascular disease, diabetes, hypertension, inflammation, allergies, cancer, renal disorders, neural function and improve immune response [5,10,11].

All fish are rich in long chain n-3 PUFA, especially EPA and DHA, but this is especially true for oily fish such as salmon and mackerel. The levels of these same PUFA are comparatively moderate in red meat sourced from pasture grazed ruminants, these having levels similar to many white fish which are low in fat such as snapper, leatherjacket, flounder [12,13]. The application of grains or some feedlot rations within livestock industries to hasten animal growth rates can diminishes the level of n-3 PUFA in red meat. In addition, recent climate variation has led to prolonged drought in some parts of the world, which diminishes the availability of n-3 PUFA rich feed sources to livestock and increases reliance on concentrate and commercial feeds that are rich in n-6 PUFA.

Collective research indicates that the evolutionary aspects of modern farming (agribusiness), selection of specialised pastures for high yield, commercially oriented crop and animal production systems, and food processing have contributed to alterations in the concentrations of n-3 and n-6 PUFA in pasture and field crops [9]. This is believed to be impacting the health and wellness of animals and humans. Indeed, the ratio of n-6 and n-3 PUFA (n-6/n-3 ratio) in human and animal diets is proposed to have been nearly 1:1 during evolutionary time, but direct interventions and climate variation has led to a shift towards a ratio closer to 20:1. This is of concern because present recommendations advise that animal and human diets should have a n-6/n-3 ratio of 1–4:1 to help maintain a balanced and healthy life [14]. The nutritionally important n-3 PUFA found in meat and other products, such as milk and non-lean edible parts of a carcass, are summarised in Table 1.

This overview aims to describe the biochemical basis of n-3 and n-6 PUFA and agricultural practices unique to the modern era that are applied for their enhancement in red meat. Special reference is made to their preservation, biological actions and recommended dosages within a human diet.

## 2. Synthesis

### 2.1. Molecular Structure of Omega-3 and Omega-6 Fatty Acids

PUFA can be classified by carbon chain length, where 20–24 carbon atoms are long chain and 26 or more carbon atoms are very long chain PUFA (FAO/WHO, 2008). Researchers (i.e., nutritionists, dietitians and biochemists) often use the ‘n minus’ term of notation to name the naturally occurring *cis* unsaturated FA, where the ‘n minus’ indicates the position of first double bond of the FA closest to the methyl end of the molecule. For example, ALA is designated as C18:3n-3 since the first double bond is present 3 carbon atoms from the methyl end, but this nomenclature does not specify the position and confirmation of remaining double bonds in the molecular structure [15]. In this system, the *cis* unsaturated FA are classified as n-3 (omega-3), n-6 (omega-6) and n-9 (omega-9). Chemical structure of n-3 and n-6 PUFA naturally available in meat and other dietary sources are shown in Table 2.

### 2.2. Biosynthesis of Omega-3 and Omega-6 Fatty Acids

ALA is the precursor (parent) FA of the n-3 family, whereas linoleic acid (LA) is the precursor FA of n-6 family. Among the four n-3 PUFA most commonly found in animal tissues (i.e., ALA, EPA, DPA and DHA), ALA cannot be synthesised by humans and animals and is therefore referred to as an essential FA [16]. Only plants can produce essential FA, and animals and humans must obtain these FA, through dietary means, for use in the synthesis of their longer chain n-3 and n-6 PUFA derivatives *viz*. EPA or DHA, or arachidonic acid (AA) [17]. In the body, the synthesis or conversion of ALA to its longer chain derivatives is controlled by many biological factors that, according to both animal and human studies, are slow and inefficient [18,19]. The conversion efficiency is not dependent on the metabolic demand of the body but is mainly determined by the amount of ALA and (interestingly) LA, present in the diet. This is because of their competitive nature whereby the same enzymes mediate ALA and LA desaturation and elongation processes.

**Figure 2:**
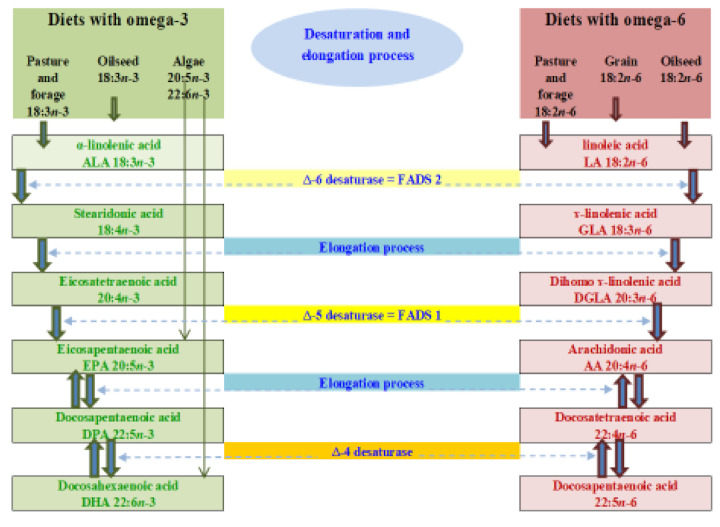
Dietary sources and biosynthesis of omega-3 and omega-6 fatty acids through enzymatic desaturation and elongation processes adapted from Ponnampalam et al. [23].

**Figure 3:**
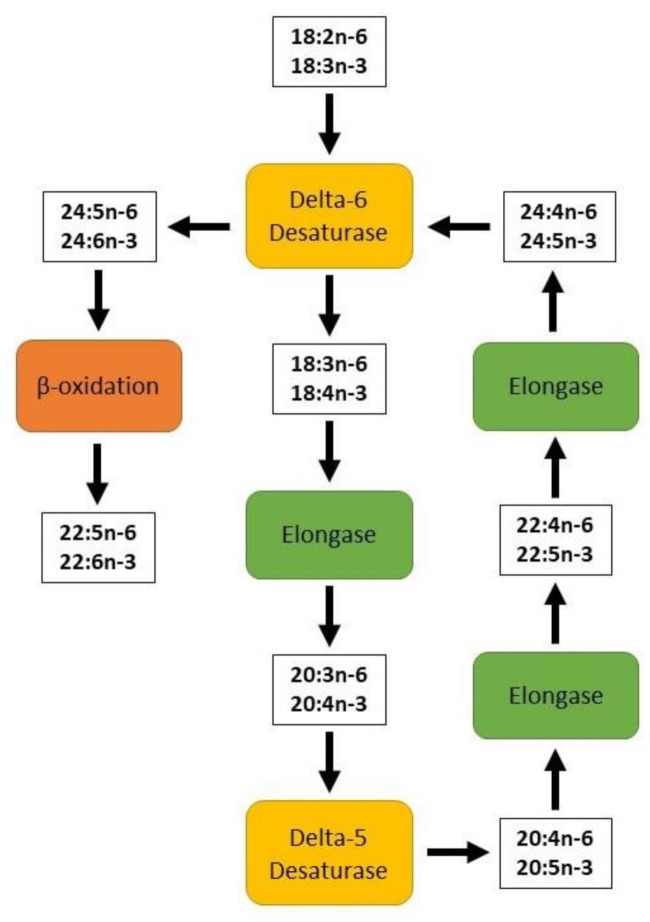
A diagram of omega-3 and omega-6 fatty acid elongation and desaturation to highlight the second use of delta-6 desaturase adapted from Gibson, Neumann, Lien, Boyd and Tu [22].

Due to the low conversion efficiency, it is necessary to provide substantial amounts of dietary ALA to promote higher levels of required EPA and DHA in the circulatory and tissue systems. Therefore, it is suggested that animals and humans should be fed with edible wild plant leaves or vegetable oils rich in ALA or, alternatively, with marine-based diets which are rich sources of EPA and DHA and avoid the desaturation and elongation processes required by ALA [20,21]. There are studies that have also shown significant increases in EPA, DPA and DHA concentrations in the blood of humans or muscle tissues of ruminants when terrestrial-based diets rich in ALA, such as flax(seed) or canola, are fed for long durations and/or at high doses [3,5,6,13,18,19,20,21,22]. Some studies indicated that there are three desaturase enzymes involved in the formation of 22 carbon long chain n-3 (DHA) and n-6 (DPAn-6) PUFA from ALA and LA in human and animal tissues. The dietary sources and biosynthetic pathways of n-3 and n-6 PUFA in mammals involving delta-6, delta-5 and delta-4 desaturase enzymes are shown below in Figure 2. It should be noted that some research has found there to be no involvement of delta-4 desaturase enzyme activity in the conversion of ALA to DHA and LA to DPAn-6, respectively. Rather, there will be a further elongation through the second use of delta-6 desaturase enzymes and then beta-oxidation processes take place in the circulatory and peripheral tissue systems for the synthesis of DHA and DPAn-6 PUFA [22]. This is illustrated in Figure 3.

Long chain PUFA (LCPUFA) in animal and human muscle tissues are mainly found in phospholipids, where they play a major role in the metabolic, functional and physiological status of the body, organelles and tissues. In vivo studies conducted in animals have indicated that the relative levels of n-3 and n-6 LCPUFA in animal tissues can be regulated by altering the balance of ALA and LA in the diet. Gibson, Neumann, Lien, Boyd and Tu [22] used rats as a model species to show that feeding ALA at 1–3% and LA at 1–2% of dietary energy, while maintaining the intake of total PUFA less than 3% of dietary energy, DHA in plasma phospholipid can be positively and linearly increased. Mammals can convert ALA into LCPUFA such as EPA, DPA and DHA via a series of desaturase and elongase catalysed reactions [24]. Both the FA desaturase 1 (FADS1) and FA desaturase 2 (FADS2) prioritise ALA compared with LA. High LA intake, such as characterised by grain finishing or feedlot feeding of animals, can interfere with the desaturation of ALA and also of 24:5n-3, which is a precursor of 24:6n-3, the final precursor of DHA (Figure 3). The concentration of ALA present in the phospholipids of plasma and tissues is usually less than 0.5%. It is not known whether this level is sufficient for FADS2 to compete with LA, which is comparatively more abundant in animal tissues [25]. Past research has indicated that the conversion of ALA to DHA is not immediate, nor as effective as direct consumption of fish or a fish oil supplement [26,27,28].

Human studies conducted using isotope-labelled ALA have shown that males, when compared to females, are less efficient at synthesising EPA and DHA from ALA. The estimated net conversion rates of ALA to EPA is 21% for females and 8% for males, and of ALA to DHA is 9% for females and 0% for males. Sex differences in EPA and DPA content have been observed, with females having higher erythrocyte phospholipid EPA, lower adipose tissue EPA and lower plasma DPA content than males. There was a significant difference between sexes in terms of human response to increased dietary ALA, with females having a significantly greater increase in the EPA content of plasma phospholipids after six months of an ALA-rich diet compared to males [29]. A detailed study of genetically divergent sheep, raised in several disparate production regions, showed there to be a small gender effect on health claimable fatty acid content EPA and DHA such that females had higher levels than males. As female lambs approach their reproductive stage, it is possible that they synthesise more n-3 PUFA in the body for the production of series-3 eicosanoids, which is associated with the ovulation process, conception and pregnancy. Lambs from Merino dams had about 2 mg/100 g higher levels of EPA + DHA than lambs from crossbred dams when the sire breed was Poll Dorset. This is similar to Ponnampalam et al. [30], who found that the ratio of PUFA to saturated FA (SFA) in meat increased from second cross Poll Dorset to first cross Poll Dorset and from first cross Poll Dorset to purebred Merino. This same study also found this to be due to an increase in PUFA, and not due to a decrease in SFA [30].

Metabolism studies using stable isotope labelling, candidate gene single nucleotide polymorphisms (SNP), genome-wide association studies (GWAS) and metabolomics show interindividual variation in the conversion of LCPUFA precursors to LCPUFA products depends on genetic factors [31]. The FA desaturase genes (FADS1 and FADS2) code for enzymes that catalyse the introduction of double bonds at specific positions in a FA chain. FADS1 (D5-desaturase) and FADS2 (D6/D8/D4-desaturase) have specificity for several FA substrates [32]. Minor allele homozygotes (D/D) had significantly lower expression of FADS1 than the I/I major allele homozygotes. ARA is the immediate product of FADS1, leading directly to the hypothesis that individuals carrying D/D genotype have lower metabolic capacity to produce LCPUFA from precursors than I/I individuals. It was reported that individuals with I/I genotype having higher metabolic capacity to convert precursors to longer chain PUFA may be at increased risk for proinflammatory disease states as they efficiently convert LA to ARA [2] as FADS SNP was found to influence synthesis of ARA and synthesis of pro-inflammatory lipoxygenase products.

## 3. Sources

Twenty and 22 carbon LCPUFA, especially ARA, EPA and DHA, are ubiquitous in mammalian tissue, are bioactive components of membrane phospholipids and serve as precursors to cell signalling eicosanoids and docosanoids that are major drug targets (e.g., COX-1, COX-2 inhibitors, leukotriene receptor antagonists). LCPUFA can be obtained directly from animal foods or endogenously synthesised from 18 carbon essential FA precursors LA and ALA and their metabolites by an alternating series of desaturation and elongation reactions [32]. Vegans rely on this biochemical pathway to generate all LCPUFA from precursors. Classic carnivores (e.g., cats and most marine fish) have lost the metabolic ability to make LCPUFA and rely on consumption of animal tissue or fish to supply all their LCPUFA requirements.

### 3.1. Feeding Type and Digestive System of Ruminants

Cattle, sheep, goats, buffalo, yak, alpacas and deer are categorised as ruminants and are unable to digest plant material directly because they lack the enzymes needed to break down cell walls (cellulose and hemicellulose). Ruminants have a complex four-chambered stomach, comprising rumen, reticulum, omasum and abomasum, due to the nature of the high roughage feedstuffs they consume. Ruminant animals support a large population of bacteria, protozoans and fungi in their four-chambered stomach because they consume a large proportion (80–85%) of highly fibrous plant materials (roughage diets). Ruminant microorganisms play a major role in the degradation of undigestible fibrous materials, thereby making use of the dietary energy and nutrients by themselves as well as providing a medium for digestion and absorption in the small (duodenum, jejunum and ileum) and large (cecum, colon and rectum) intestines of the host animals. The important function of the salivary gland is adding saliva to the feeds to form bolus and to buffer pH levels in the rumen and reticulum so that the microbial activity and degradation process is optimised. The rumen and reticulum are home for the population of microorganisms that ferment and break down plant materials and produce volatile organic compounds and release other nutrients—both microbes and host animals use these volatile organic compounds for energy. The anatomical and functional attributes of small intestine of ruminants is similar to non-ruminants and ranges in length between approximately 12–30 times the body length of the animal [33].

### 3.2. Digestion, Absorption and Deposition of Dietary Lipids in Tissue of Livestock

Lipids are either consumed or synthesised de novo to contribute structure, integrity, recognition systems and energy to cells of most tissues. Not many researchers realise that the digestion and absorption of lipids (or fats) in ruminant and monogastric animals are different. This is due to their feeding nature and structure of digestive systems. In general, diets consumed by ruminants consist of 80–85% roughage and 15–20% concentrate while the diets consumed by monogastric animals are the opposite. More details on digestion, absorption and metabolism of dietary lipids can be found elsewhere [33,34]. Ruminant diets generally consist of 1–4% fat, and lipid supplements fed to ruminants above 5–6% on a dry matter basis have negative effects on rumen microbial activity, mainly on carbohydrate (fibre as cellulose and hemicellulose) degradation, particularly when PUFA are included in the diet. Supplementation of lipids in ruminant diets have some benefits to livestock industry in the following aspects: (1) it helps reducing the methane emission to environment from degradation of high fibrous diets; (2) it helps bypassing the dietary lipids (PUFA) from rumen to small intestine for absorption avoiding biohydrogenation; and (3) saving the dietary energy captured from methane emission for assimilation of tissue growth. With monogastric animals having a stomach as one organ for temporary storage of diet (fats) in the absence of rumen microbial activity, they can handle greater amounts of lipids in their diet for digestion and absorption process.

In any species, acetate (mainly cattle and sheep as ruminants) or glucose (mainly swine and poultry as monogastric animals) is absorbed in the intestine to enter FA biosynthesis via malonyl-CoA production through the acetyl-CoA carboxylase reaction and then palmitate production through FA synthase. Once palmitate is synthesised, other medium to long chain SFA and MUFA are generated by desaturation and elongation process. Since animals cannot synthesise essential PUFA (ALA and LA), these lipids have to come from consumed feeds. In monogastric animals, dietary fats are unchanged by digestion in the intestine so that tissue FA more directly reflect their present in the diet. Several steps are involved in resynthesis and transport of lipids in ruminants from the enterocyte where FA are absorbed, until they reach the peripheral tissues such as adipose and muscle tissues. The FA, monoglycerides and diglycerides reaching the jejunum from micelles are absorbed into the epithelial cells of small intestine. These FA are esterified, and triglycerides and phospholipids are assembled into lipoprotein particles (chylomicrons, very low-density lipoproteins, etc.) in the enterocyte, which are then secreted into lymph vessels and enter the bloodstream.

**Figure 4:**
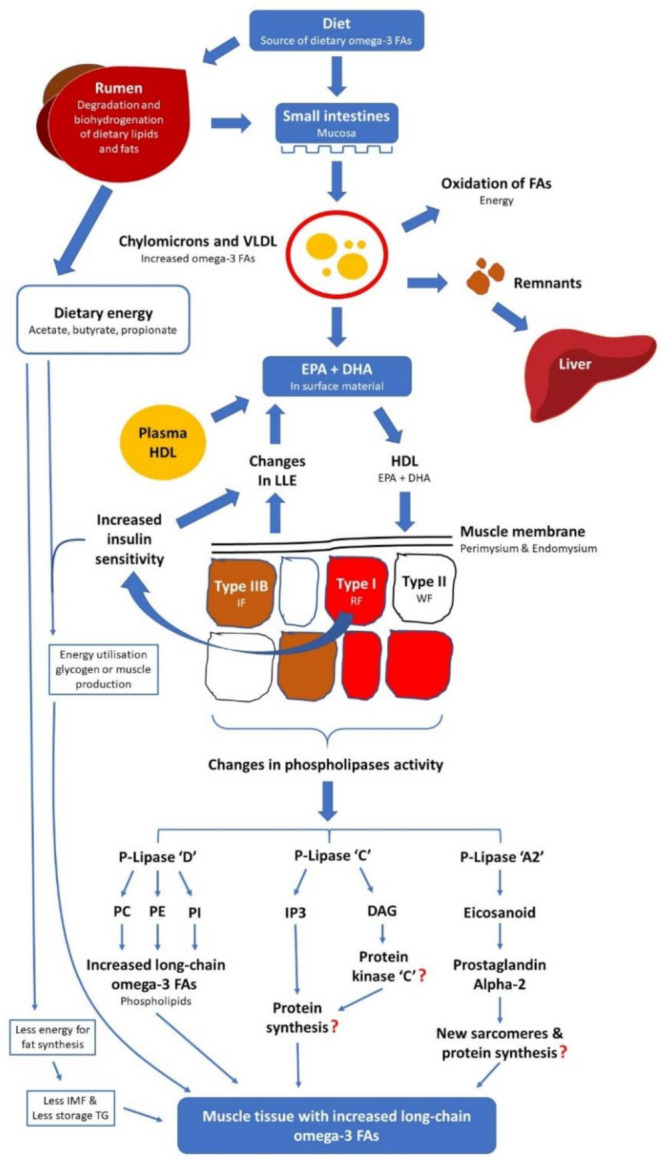
The mechanisms of dietary omega-3 fatty acid digestion and metabolism in ruminants, adapted from Ponnampalam [34]. Abbreviations include fatty acid (FA); very low-density lipoproteins (VLDL); eicosapentaenoic acid (EPA); docosahexaenoic acid (DHA); high-density lipoproteins (HDL); lipoprotein lipase enzyme (LLE); intermediate fibres (IF); red fibres (RF); white fibres (WF); phosphatidylcholine (PC); phosphatidylethanolamine (PE); phosphatidylinositol (PI); Inositol triphosphate (IP3); diacylglyceride (DAG); and triglyceride (TG or triacylglycerol (TAG)).

In monogastric animals, the liver plays a major role in FA synthesis. In ruminant animals, the contribution of liver is minimal and, instead, FA synthesis is very extensive in adipose tissue. Upon entry to the blood, chylomicrons and very low-density lipoproteins acquire apoproteins apo-C and apo-E provided by high-density lipoprotein. Apo-C inhibits liver removal of chylomicrons and very low-density lipoproteins and this enhances the extent of diversion of these entities to other tissues. One of the apo-C components activates the lipoprotein lipase enzyme, which is situated primarily on the surface of the endothelium of skeletal muscle, adipose and mammary tissue sites. FAs and partial glycerides are apportioned to triglycerides, phospholipids and other lipids in the organs or oxidation for energy according to the metabolic demands of the body either in skeletal muscles, adipose and/or mammary tissues. The state of dietary lipids rich in n-3 PUFA from digestion in the intestine to deposition in the peripheral tissues through the circulatory systems is shown in Figure 4.

In the circulatory or tissue systems, diacylglycerol (DAG) is produced from phosphatidylcholine or from other phospholipids. Phospholipase C (PLC) cleaves the membrane phospholipid phosphatidylinositol-4,5-bisphosphate (PIP_2_) to generate inositol-1,4,5-trisphosphate (IP_3_) and DAG. Another type of phospholipase, phospholipase D (PLD), is activated by various stimuli in the cell. PLD hydrolyses phosphatidylcholine, which is abundant in the cell plasma membrane, producing phosphatidic acid and choline. Phosphatidic acid is hydrolysed by phosphatidic acid hydrolase to release DAG and phosphate. This is a second pathway that generates DAG. While this intermediate is the product of the action of both PLC and PLD, cellular responses in both cases are usually not identical due to differences in the cellular localisation of enzymes or the fatty acid composition of the DAG produced. The stimulation of specific cell-surface receptors activates phospholipase A2, leading to the release of arachidonic acid from the cell membrane.

### 3.3. Factors Affecting Omega-3 and Omega-6 Fatty Acid Deposition in Muscle Tissues (Meat) of Ruminants

The modern era has brought advancement in agricultural practices and food processing. However, large scale commercially oriented crop and animal production has, in general, decreased n-3 PUFA concentrations, increased n-6 PUFA concentrations and increased n-6/n-3 ratios in meat, eggs and milk when sourced from intensively farmed animals compared to animals living in range lands. Within this context, there are three things to recognise. The amount of a specific n-3 PUFA deposited in the cells and tissues (1) are not directly related to the amounts of n-3 PUFA present in the animal diets; (2) are related to the amount of n-6 PUFA available in the feeds for consumption and amount already deposited in the peripheral tissues; and (3) are dependent on the interference of desaturation and elongation enzymes reactions of both FA families that generate their LCPUFA in tissues. Many different factors influence the n-3 and n-6 PUFA concentrations of meat in ruminants, which will be briefly discussed below.

A. Type of based diet: It has been well established that pasture feeding systems or fodder feeds containing silage produce meat with more n-3 PUFA than the grain feeding or lot feeding system. Green leafy materials contain more ALA in the chloroplasts, whereas the grain-based or cereal-based diets often contain more LA and MUFA. For example, a high concentration of LA and AA is found in meat from ruminants consuming grain-based feeds in contrast to ruminants consuming brassica-based feeds or lucerne (alfalfa) pasture-based feeds which have more ALA, EPA and DHA in their meat [35].
B. Type of supplement (ingredient) used in the diet: Supplementation of oilseeds and meals from sunflower, safflower, corn and cotton to ruminant diets could elevate n-6 PUFA whilst flax, canola, chia and camelina can increase the concentration of n-3 PUFA within meat [36]. LA is found in most plant and cereal seeds and is abundantly available naturally for ruminant consumption. Therefore, it is expected that the concentration of n-6 PUFA would be greater than n-3 PUFA and lead to a higher n-6/n-3 ratio in the meat from these animals.
C. Form of lipid present in the diet: It has been reported that the efficacy of direct absorption of product FAs (AA, EPA and DHA) were greater than parent FA (LA and ALA). For example, when EPA and DHA were directly consumed by ruminants as algae or fish oil supplementation, the deposition of EPA and DHA were greater than when feeding diet containing ALA (flaxseed) to increase the concentration of EPA and DHA within cells and tissues [6,20,21], and the same outcomes can be applied to other n-6 PUFA. Moreover, the ratio of EPA, DHA and ALA to the total PUFA in the diet can determine the amounts of EPA, DHA or ALA absorbed at the enterocyte and further deposition at the peripheral tissue sites [22].
D. Competition between desaturation and elongation enzymes: The affinity of FADS2 for ALA is greater than LA. Nevertheless, high intake of LA or high concentration of dietary LA can adversely affect the conversion of ALA within tissues. The nature of the current animal production systems prefers the application of concentrate feeding for fast growth and quick turn over to market, offering large quantity of dietary LA available for animal consumption and this results in greater deposition of LA and AA than their counterparts of ALA and its derivatives EPA, DHA or DPA [37]. A recent study indicated that the application of forages, such as lucerne hay around 50%, in the mixed ration, compared with a ration containing 50% of grain made of barley and oats, significantly altered the concentration of ALA, LA, and EPA of muscle fat in two sheep types of diverse genetics namely pure Merino and Crossbred sheep. This same study showed a diet by breed interaction that was proposed to be the result of different concentration of ALA and LA being deposited in muscle fat of those two genetics, allowing for domination of one type of FA on another when deposited at or above certain concentrations in the muscle fat [38]. When the animals were fed lucerne hay diet, the crossbred lambs produced higher ALA in the tissues and had 20 mg higher ALA/100 g tissue than the Merino lambs (ALA concentrations for Crossbred and Merino animals were 50 vs. 31 mg/100 g muscle). This was not observed in animals fed the grain-based diet, and the ALA concentrations for crossbred and Merino animals were 30 vs. 15 mg/100 g muscle. The greater ALA concentration in muscle tissues from crossbred lambs fed the lucerne hay diet might have suppressed the LA deposition whilst enhancing the elongation process of ALA to its longer chain EPA. The LA concentrations for crossbred and Merino animals fed lucerne hay diet were 103 vs. 95 mg/100 g muscle while the values for grain fed sheep were 168 vs. 138 mg/100 g muscle, respectively.
E. Availability of secondary metabolites: Animal feeds contain a vast range of secondary metabolites called phytonutrients. Pastures, fodder crops and higher plant species produce (secrete) these phytonutrients in their body for their protection, survival and establishment against disease, pest and harsh climate under various conditions. These phytonutrients have health enhancing compounds and animals may selectively consume these pastures, fodders and other by-products of field crops to support their good health and wellbeing. Phytonutrients are classified as alkaloids, polyphenols, organosulfur compounds and so on. It is likely that animal feeds containing some types of polyphenols, such as tannins, phenolic acids and flavonoids, can protect dietary PUFA from the hydrolysis and biohydrogenation in the rumen resulting in beneficial effects. Hence, increased n-3 LCPUFA would be available for absorption across enterocytes and, therefore, have increased deposition within tissue and meat. This is possibly due to these phytonutrients having low bioavailability and long retention times within the rumen, causing a slow degradation of fibrous diets by the microflora and allowing the PUFA and other nutrients present in the diet to bypass the rumen and be available for intestinal absorption by host animals.
F. Level of antioxidants and carotenoids in muscle tissues: Ruminants are specialised to consume 80–85% of diet as forage (fibrous materials) such as green pastures, fodders, silage and other forage materials. From these diets, they ingest adequate amounts of antioxidants, such as vitamins, minerals and carotenoids. Monogastric animals grown under intensive systems consume 80–85% concentrated diets and they receive carotenoids and antioxidants from the ingredients of cereal grains, protein meal and oilseeds. Carotenoids, such as carotenes and xanthophylls, are pigments present in leaves, seeds, fruits and animal products of blood, meat and milk. Carotenoids have the ability to act as antioxidants as they are quenchers of singlet oxygen (^1^O_2_) and other reactive oxygen species (ROS) or substances that causes oxidative damage in the body from cell to tissue level. The biological roles of carotenoids and polyphenols in the ruminant digestive system and their metabolism are not yet fully understood. It is speculated that increased level of antioxidant potential in the circulatory and tissue systems can protect the oxidation of n-3 LCPUFA from the tissues. This improves the health and wellbeing of individuals. For example, several studies [7,21,35,36,37] described the relationship among antioxidants, n-3 PUFA and lipid oxidation in muscle tissues in sheep and goats.

## 4. Biological Actions

In the context of human health and wellness, ALA, EPA, DPA, DHA and their secondary metabolites have been the focus of attention throughout the previous 50 years. It seems reasonable to suggest that the mode of actions and effects would be similar between farm and companion animals. The dramatic advancement in the analytical technologies of n-3 PUFA, identification of their intermediate metabolites and understanding of their important role in human growth, development and disease prevention has facilitated the introduction of new dietary regulations and recommendations for foods high in PUFA and *trans* FAs, particularly n-3 PUFA and vaccenic acid. In this context, long chain PUFA are considered essential and/or health enhancing nutrients that impact on growth and development in early life as well as metabolic disorders and chronic diseases in later life.

**Figure 5:**
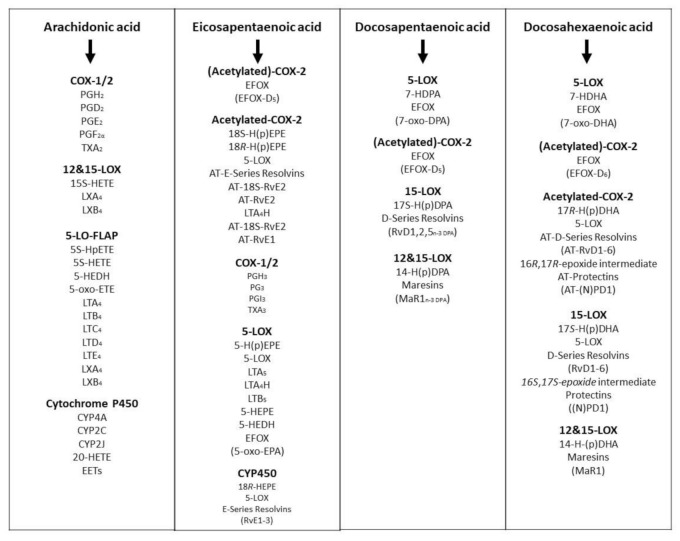
The formation of lipid mediators (intermediary metabolites and isoforms) from eicosanoids and docosanoids derived from long chain omega-3 (EPA, DPA, DHA) and omega-6 (AA) fatty acids in animals and human tissues or body.

DHA is a major constituent of cardiomyocytes, sperm, grey matter of the brain and the retina. Several studies have indicated DHA is necessary for central nervous system functionality as well as the visual activity of infants. The 20 and 22 carbon chain-length PUFA (i.e., EPA, DHA, and AA) can be converted to a series of hormone-like substances called eicosanoids and docosanoids, respectively, including prostaglandins (PGs), thromboxanes (TXs), prostacyclin (PGI2), leukotrienes (LTs), resolvins (RVD) and other lipid mediators (Figure 5). These eicosanoids and docosanoids contain many intermediary metabolites and isoforms. These agents play major roles in the regulation of diverse pathophysiological functions, including blood pressure, platelet aggregation, blood clotting, blood lipid profiles, immune response, the inflammation response to injury and infections and the resolution of inflammation [15,39]. A large proportion of research conducted in laboratory animals and humans has been devoted to the pathophysiological functions and properties of EPA, DPA and DHA and the roles of the derived lipid mediators.

The ARA is then rapidly converted into two major classes of enzymes, called cyclooxygenases (COX) and lipoxygenases (LOX). COX enhance the production of prostaglandins, prostacyclin and thromboxanes, while lipoxygenase enhance the production of leukotrienes. The physiological actions of these metabolites are widespread and diverse. Briefly, prostaglandins and prostacyclin are potent vasodilators whilst thromboxanes are potent vasoconstrictors, whereas leukotrienes produce bronchoconstriction. Lipoxygenases in plants and animals are heme-containing dioxygenases that oxidise PUFA at specific carbon sites to give enantiomers of hydroperoxide derivatives with conjugated double bonds. The number in specific enzyme names such as 5-LOX, 12-LOX, or 15-LOX refers to the ARA site that is predominantly oxidised. Of these, 5-LOX is best known for its role in the biosynthesis of leukotrienes A4, B4, C4, D4 and E4. The oxidised metabolites generated by 5-LOX were found to alter the intracellular redox balance and to induce signal transduction pathways and gene expression. The enzyme 5-LOX has been identified as an inducible source of ROS production in lymphocytes [40]. Cyclooxygenase-1 has been implicated in ROS production through formation of endoperoxides, which are susceptible to scavenging by some antioxidants in cells stimulated with TNF-α, interleukin-1, bacterial lipopolysaccharide, or the tumour promoter 4-otetradecanoylphorbol-13-acetate [41].

DPA is another n-3 LCPUFA which has potential in maintaining health and wellness of animals and humans. Its applicability and efficacy in terms of metabolic activity and disease prevention have not been fully investigated. However, it deserves attention for various reasons, including that it is the intermediate substrate of EPA conversion to DHA within the cell or tissue systems of animals and humans, and its tissue concentration is dependent on the balance between EPA and DHA. There is emerging evidence that DPA levels are positively correlated with the expression of certain enzymes involved in inflammatory processes of the cardiovascular system [42]. Research indicates that DHA and its metabolites are used for tissue-based metabolic activities such as insulin-stimulated energy disposal, phospholipid-induced signal transduction towards gene expression, active autoimmune systems towards cell defence, etc. Within these contexts, the overall n-3 PUFA metabolic process must be efficient in converting EPA to DHA via DPA and the reverse reaction of DPA to EPA; and the availability of DPA in the tissue system is important for transitional processes to maintain both EPA and DHA levels. It is noteworthy that the concentration of DPA in red meat is equal or greater than that of EPA and DHA. Therefore, the contribution of DPA from red meat should not be neglected or ignored in terms of its role in the maintenance of health and wellness of people who regularly consume more red meat than fish or vegetables.

## 5. Dietary Recommendation

Past research has determined adults (aged 18 years and older) to have no upper intake limit for n-3 PUFA to ensure their safety, *viz.* ALA, EPA, DHA and DPA [43,44]. Instead, it is apparent that diets which fail to provide the minimum requirements of these FA are a greater health concern. In response, many authorities and organisations from around the world have proposed guidelines that define the daily recommendable intakes for n-3 PUFA, n-6 PUFA and total LCPUFA (Table 3). From these, we can observe that male and female adults have different requirements and, furthermore, the requirement for females will depend on their physiological status (e.g., every day, during pregnancy, during lactation). This complements previous knowledge that age will impact on dietary requirements for these PUFA, with children aged less than 18 years proposed to require a diet that contains more n-3 PUFA and n-6 PUFA than necessary for an adult [45].

The examples included in Table 3 also show that dietary FA guidelines differ between organisations and, sometimes, these differences are substantial. A possible basis for this disparity could be the basal diets typical to the populations represented by these organisations. For example, MHLW [49] identify the diet of the Japanese population to be comparatively lower in n-6 PUFA and, therefore, a lower requirement for n-3 PUFA is necessary to achieve an acceptable n-6/n-3 ratio. This is reliant on the n-6/n-3 ratio’s importance to human health, an observation previously made and resulting in ratio recommendations that range from 5:1–10:1 for adults [46]. That said, the FAO [54] report that if n-3 PUFA and n-6 PUFA intakes adhere to their individual guidelines then there is no rationale to support a recommendation for n-6/n-3 ratio intake. This premise does depend on their being no biochemical competition or inhibition between the functionalities of n-6 PUFA and n-3 PUFA that affects their bioavailability.

## 6. Concentration Range in Red Meats

Approximately 80% of Australians are not meeting the recommended n-3 LCPUFA intake for optimum health [55]. The same is likely true for majority of the population in other Western countries such as United States of America and the United Kingdom. The general advice from dieticians and health professionals is to consume 2–3 fish meals weekly to elevate the n-3 LCPUFA levels in the body. Fish and other seafood are rich sources of n-3 LCPUFA. Nevertheless, the consumption of LCPUFA (i.e., EPA and DHA) from marine-based foods is low to very low in many Western countries, and alternative food sources for these FA may therefore be advantageous in these populations.

Unlike human and other monogastric animals, ruminants are mainly dependent on the microbial population in the rumen for the digestion and absorption of dietary lipids. This microbial activity is responsible for the hydrolysis of dietary lipids and further isomerisation and conversion of unsaturated FAs into MUFA and SFA intermediates. The latter process leads to an increase in stearic acid (C18:0) concentration for small intestine absorption. However, several studies have reported that PUFA content in red meat from ruminants can be significantly modified by feeding systems. Pasture- and silage-fed animal deliver meat with higher PUFA, particularly in terms of n-3 PUFA content, when compared with their grain-fed and concentrate feedlot fed counterparts [56,57,58]. Particular secondary metabolites found in pasture and forage diets may exert a greater protection against microbial biohydrogenation of PUFA in the rumen and, therefore, facilitate increased absorption and deposition of PUFA in ruminant tissues [59]. Previous research has also shown considerable differences on animal growth performances, carcass characteristics and meat quality attributes in sheep and cattle fed forage-based diet versus concentrate diet and these areas have been discussed in detail by others [60].

Western populations consume more meat and processed meat products than marine-based foods due to preference, availability and affordability. As a consequence, red meat can contribute up to 20% of their n-3 LCPUFA requirements [61]. An earlier study in humans consuming lean red meat showed that 2 weeks consumption of 500 g lean meat per day was sufficient to raise plasma DPAn-3 levels [62]. This may therefore be considered an alternative or complementary source for those with poor fish consumption. The enrichment of n-3 PUFA levels in meat through dietary management has been a focus in the animal production systems for the past 20 years. This aims to improve n-3 PUFA consumption for those who consume lower amounts of meat [20,34,63,64]. For this reason, many studies have investigated feeding lipid sources such as marine-based oils, and grains and oilseeds in ruminant and monogastric animals. To our knowledge, the studies conducted with algae, fish oil, flaxseed and canola seed supplementation and, likewise, using specialised forage or grazing options have shown prominent outcomes in increasing the n-3 LCPUFA levels in ruminants [65,66,67,68,69]. This information provides insight into management practices that can optimise the nutritional value of the meat products. That said, ALA is the primary FA source from plant-based diets. A good understanding of the efficacy of the elongation process of ALA to EPA, DPA and DHA in ruminants is long overdue [17].

## 7. Preservation until the Point of Consumption

Management systems have been adopted that enrich animal tissues so that they become a source of n-3 PUFA and n-6 PUFA. These efforts are often implemented without first considering the interim between processing and consumption. This is important as longer carbon chain-length FA with double C-H bonds (e.g., EPA or DHA or AA) are more susceptible to oxidation than shorter or more hydrogenated FA, such as MUFA [70,71]. Manifestations of this effect are observed in Adeyemi et al. [72], with results showing that n-3 PUFA and n-6 PUFA concentrations in goat meat declined across 12 days of a chilled storage period at 4 °C; in Muino et al. [73], with findings that lamb PUFA decreased in a linear trend with increased chilled storage period when held in oxygen-rich modified atmospheric packaging; and in Diaz et al. [74], with conclusions that 6 days of chilled storage at 2 °C was sufficient to degrade the PUFA content of lamb meat. It is interesting, therefore, that Holman et al. [75] observed no change in beef PUFA composition across a 12 week chilled storage period—although the authors suggest this was an outcome of anaerobic storage, low initial levels of PUFA and a relatively high concentration of vitamin E within the samples.

Vitamin E (α-tocopherol) has been widely acknowledged as practical and intrinsic means to preserve the FA profile of meat. Indeed, for lamb meat, Ponnampalam et al. [76] proposed a tissue concentration of greater than 3.45 mg/kg vitamin E as sufficient to inhibit excessive peroxidation. A similar recommendation was made for beef, with Arnold et al. [77] concluding that concentrations of 3.3 mg/kg vitamin E were appropriate. The concentrations of tissue vitamin E have been reportedly improved with animal supplementation [78,79] and functions as a result of its inhibition of the production of reactive oxygen species and propagation of free radical reactions [80]. Alternatively, meat may be stored within anaerobic packaging conditions or with embedded antioxidants to inhibit peroxidation. Examples have been described in Holman et al. [81] with review of different patents for smart packaging devices and antioxidant coatings that can scavenge specific gases, including oxygen, from an in-pack atmosphere to preserve against oxidation, and can be implemented within packaging systems to assure anaerobic conditions. That said, temperature controls (cold-chain, frozen storage) and vacuum packaging alone may be enough to prevent excessive peroxidation if their consistency and efficacy can be confirmed across the interim. From these findings, it is recommended that the preservation of FA composition beyond its immediate enhancement should be considered when seeking to enrich the composition of different food types.

## 8. Conclusions

We report here that animal and human foods in the modern era are composed of higher n-6 PUFA levels and n-6/n-3 ratio compared to foods consumed by humans and animals during early evolutionary periods. These variations are primarily the consequence of changes to agricultural practice, animal production systems and food processing during the last 100–200 years. Changes in ecosystems and climate variability also contribute to these variations. Taking Australian production systems as an example, it is perceived that the application of low-nutritive or low-quality roughage diets (haylage), crop residues, senesced hay materials in the ruminant production systems is vital for sustainable and resilient future animal industries, but this will further reduce the n-3 PUFA and vitamin consumption and as a consequence in red meat. Commercially based animal industries using proportionately high concentrate diets in their animal feeds may also be attributable to increased consumption of n-6 PUFA and reduced n-3 PUFA by livestock and, thus, elevated n-6/n-3 ratio in red meat. It is likely that animals grazing single stand pasture (monoculture) receive lower amounts of essential FA, vitamins and minerals than those grazing mixed pastures. This is due to limited selection of herbage materials that are rich in nutrient values. It is known that ruminants consuming feeds rich in lipids mainly n-3 PUFA (oils and fats—e.g., diets containing brassica family members such as canola, camelina, or flax) also emit lower amounts of methane to the ecosystem than those consuming diets with highly fibrous structural carbohydrates, such as diets high in cellulose. Forages high in secondary metabolites such as polyphenols (tannins, flavonoids and phenolic acids), alkaloids and carotenoids may protect n-3 PUFA against microbial fermentation and biohydrogenation in the rumen due to their low bioavailability, allowing PUFA to reach the intestine for the absorption by host animals. This observation notwithstanding, additional research is necessary in this area to better understand the biological pathways and mechanisms of actions.

A sustainable animal and plant production system is essential for economic viability and the health and welfare of animals and humans, reinforcing the consideration of n-3 PUFA and n-6 PUFA in animal feeding systems equivalent to range feeding. The literature clearly indicates that animal grazing diets high in essential FA and vitamins have better metabolic conditions and oxidative status than those consuming diets of low nutritive value, contributing to improved wellness and lower veterinary care. It is likely that ruminant animal feeding systems will, in the future, utilise more concentrate-based specialised diets, which consist of less n-3 PUFA, to tackle the extended dry seasons and shortage in green pasture with climate variation. This scenario requires that the producers and researchers identify forage diets and supplements high in n-3 PUFA, vitamins and phytonutrients whilst low in n-6 PUFA and structural carbohydrates (cellulose, lignin) so that the health and wellbeing of animals and humans can be advantaged. The capacity to maintain the essential PUFA, vitamins and trace elements in meat from farm to fork and throughout processing and preservation must also be considered. Taken together, we state that offering n-3 PUFA rich diets to animals has many advantages economically, environmentally and socially, not only for animals but also for those humans who consume red meat in moderate to high quantities.

## Figures and Tables

**Table 1:** Common name, abbreviation and scientific name (IUPAC, International Union of Pure and Applied Chemistry) of omega-3 (n-3) and omega-6 (n-6) fatty acids found in dietary sources.

Common Name	Abbreviations	Systematic Name
Linoleic acid	C18:2n-6 (LA)	cis-9, cis-12-octadecatrienoic acid
Alpha-linolenic acid	C18:3n-3 (ALA)	cis-9, cis-12, cis-15-octadecatrienoic acid
Stearidonic acid	C18:4n-3 (SDA)	cis-6, -9, -12, -15-octadecatetraenoic acid
Arachidonic acid	C20:4n-6 (AA)	cis-5, -8, -11, -14-eicosatetraenoic acid
Eicosapentaenoic acid	C20:5n-3 (EPA)	cis-5, -8, -11, -14, -17-eicosapentaenoic acid
Adrenic acid	C22:4n-6 (AdA)	cis-7, -10, -13, -16-docosatetraenoic acid
Docosapentaenoic acid	C22:5n-3 (DPA)	cis-7, -10, -13, -16, -19-docosapentaenoic acid
Docosahexaenoic acid	C22:6n-3 (DHA)	Cis-4, -7, -10, -13, -16, -19-docosahexaenoic acid

**Table 2:** Names and chemical structures of commonly available omega-3 and omega-6 fatty acids.

Common Name	Chemical Structure
Linoleic acid	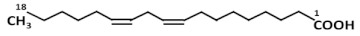
Alpha-linolenic acid	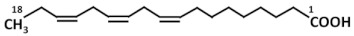
Stearidonic acid	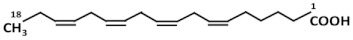
Arachidonic acid	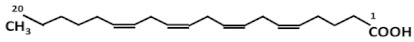
Eicosapentaenoic acid	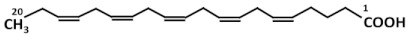
Adrenic acid	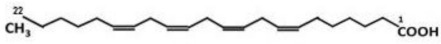
Docosapentaenoic acid	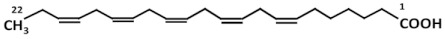
Docosahexaenoic acid	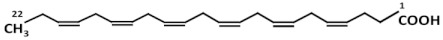

**Table 3:** Examples of omega-3 (n-3), omega-6 (n-6), and total long chain polyunsaturated fatty acid (LCPUFA) dietary recommendations for a healthy adult (18 years and older). Abbreviations include male (M); female (F); alpha-linolenic acid (ALA); linoleic acid (LA); percentage total energy (%E); eicosapentaenoic acid (EPA); docosahexaenoic acid (DHA); and acceptable macronutrient distribution range (AMDR). Please note that for female adults, recommendations are categorised using physiological status (as everyday, during pregnancy and during lactation).

Organisation	n-3	n-6	LCPUFA
NHMRC [46] Australia and New Zealand	M: 1.3 g/day ALA F: 0.8|1.0|1.2 g/day ALA	M: 13 g/day LA F: 8|10|12 g/day LA	M: 160 mg/day LCPUFA F: 90|110–115|140–145 mg/day LC-PUFA
Health Canada [47] Canada	M: 1.6 g/day ALA F: 1.1|1.4|1.3 g/day ALA	M: 14–17 g/day LA F: 11–12|13|13 g/day LA	-
EFSA [44] Europe Union	4.0%E	0.5%E	250 mg/day EPA + DHA +100–200 mg/day DHA (during pregnancy or lactation)
[48] France	0.8%E ALA	2.0%E LA	Min. physiological requirement: DHA 0.1 E%
MHLW [49] Japan	M: 2.0–2.4 g/day (total) F: 1.1|1.4|1.3 g/day (total)	M: 8–11 g/day (total) F: 7–8|9|9 g/day (total)b	
MVO [50] The Netherlands	1%E ALA	2%E LA	450 mg/day EPA + DHA
Bartrina and Majem [51] Spain	1–2%E ALA	5%E LA	500–1000 mg/day EPA + DHA
Otten [52] United States of America	5–10%E	20–35%E	10%E EPA + DHA
Food and Nutrition Board [53] United States of America	M: 1.2–1.6 g/day ALA F: 1.1–1.2|1.3|1.3 g/day ALA	M: 14–17 g/day (total) F: 21–26|28|29 g/day (total)b	2 g/day U-AMDR
FAO [54] United Nations	0.5%E ALA	2.5%E LA	-
